# Supplementary material for: Orexin neurons inhibit sleep to promote arousal
Source: Nat Commun. 2022 Jul 18;13:4163. doi: 10.1038/s41467-022-31591-y (PMC9293990; doi:10.1038/s41467-022-31591-y)
Supplement: Supplementary file 1 — Supplementary Information [file 41467_2022_31591_MOESM1_ESM.pdf]

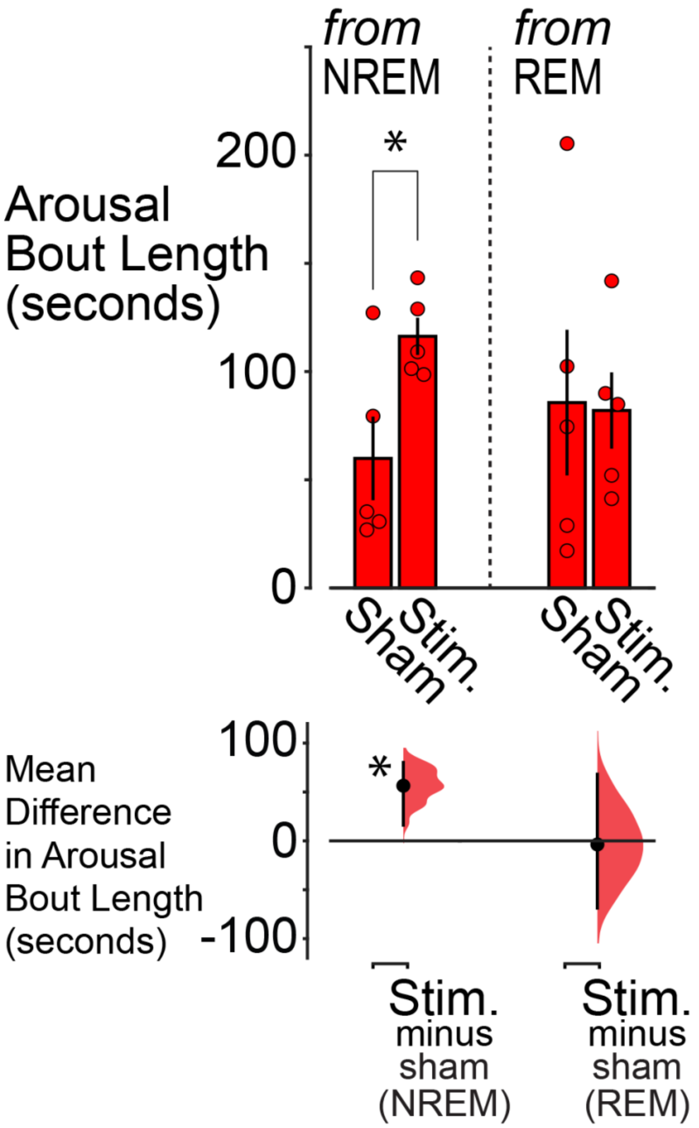

Suppl. Fig. 1. Bout length of arousals during stimulations of the orexin terminals in the VLPO. Mean length of arousal bouts initiated within the stimulation trials (sham vs stimulation at all frequencies). The effect of lengthening arousals that doubled relative to those from sham trials, was specific to NREM sleep (t=2.87, p=0.045; paired t-test; n=5). Raw data is plotted on the upper axes with mean ± SEM. On the lower axes, mean differences (sham vs stimulation within the ChR2-mCherry group) are plotted as bootstrap sampling distributions. Each mean difference is depicted as a dot. *, p<0.05 two-tailed paired t-test and n refers to the number of independent animals. Source Data are provided as a Source Data file.

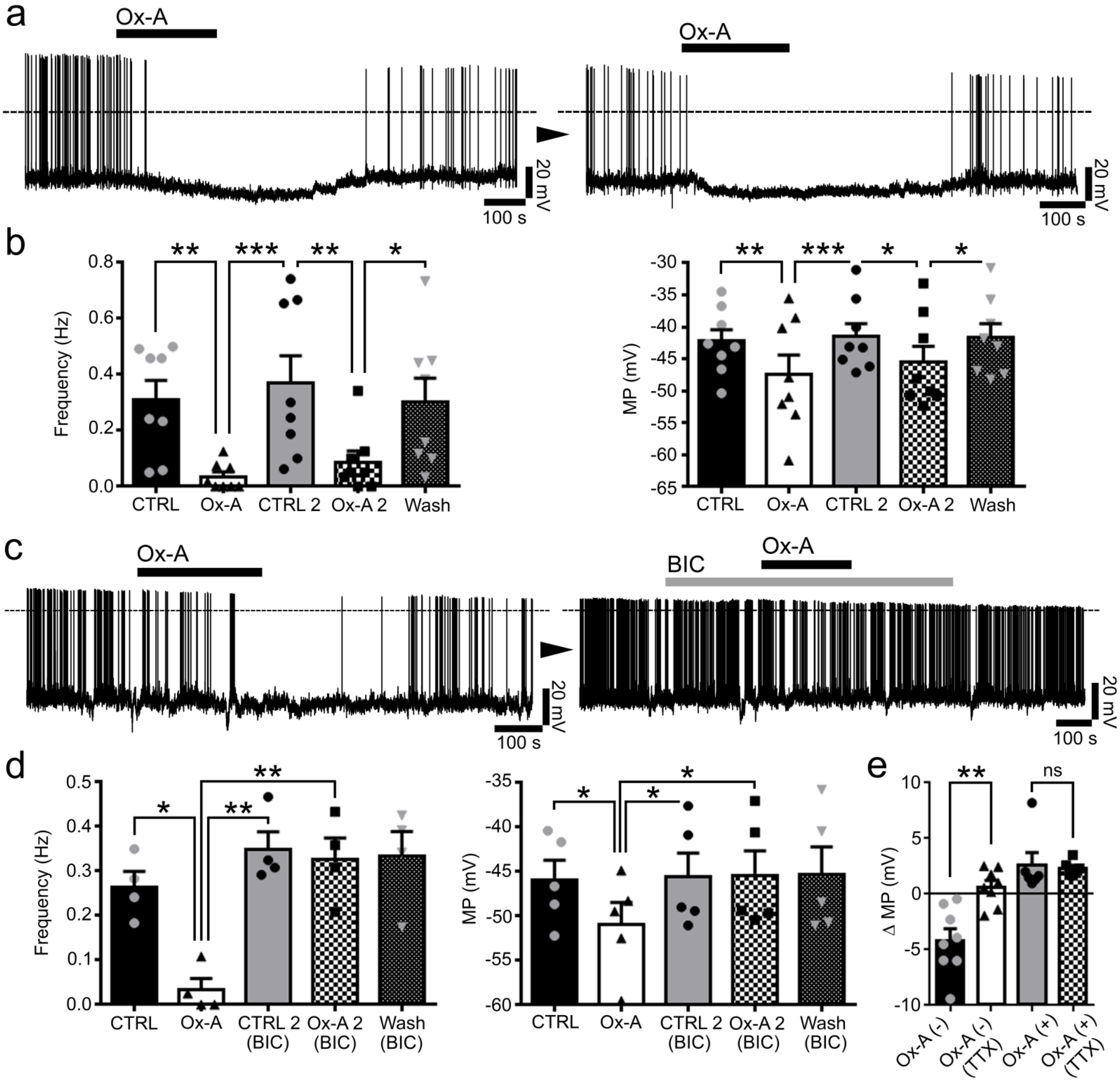

Suppl. Fig. 2. Orexin acts in VLPO by direct excitation and feedforward inhibition. a-b) Repeated applications of Ox-A inhibit VLPO neurons (*a*). Ox-A effects on the firing frequency (*b left; n*=8; one-way ANOVA, $F_{(4,\ 35)}$=10.55; *p*<0.0001; CTRL vs Ox-A, *adj-p*=0.0022; Ox-A vs CTRL 2, *adj-p*=0.0002; CTRL 2 vs Ox-A 2, *adj-p*=0.0017; Ox-A 2 vs Wash, *adj-p*=0.0263) and the membrane potential (*b right; n*=8; one-way ANOVA, $F_{(4,}$

$_{35)}$=10.94; p<0.0001; CTRL vs Ox-A, *adj-p*=0.0010; Ox-A vs CTRL 2, *adj-p*=0.0001; CTRL 2 vs Ox-A 2, *adj-p*=0.0128; Ox-A 2 vs Wash, *adj-p*=0.0204). **c-d**) Orexin-mediated inhibition of VLPO neurons is blocked by bicuculline (*c;* BIC, 20µM). Ox-A before and in the presence of BIC on firing frequency (*d left*; *n*=4; one-way ANOVA, $F_{(4, 15)}$=10.04; p=0.0008; CTRL vs Ox-A, *adj-p*=0.0194; Ox-A vs CTRL 2 (BIC), *adj-p*=0.0017; Ox-A vs Ox-A 2 (BIC), *adj-p*=0.0030) and membrane potential (*d right*; *n*=5; one-way ANOVA, $F_{(4, 20)}$=5.34; p=0.0063; CTRL vs Ox-A, *adj-p*=0.0375; Ox-A vs CTRL 2 (BIC), *adj-p*=0.0234; Ox-A vs Ox-A 2 (BIC), *adj-p*=0.0185). **e**) The Ox-A inhibitory effect (Ox-A(-)) is blocked by TTX (1µM; *n*=8) whereas the Ox-A excitatory effect (Ox-A (+)) is maintained (*n*=6; one-way ANOVA, $F_{(3, 24)}$=14.16; p<0.0001; Ox-A (-) vs Ox-A (-) (TTX), *adj-p*=0.0016 and Ox-A (+) vs Ox-A (+) (TTX), *adj-p*>0.9999). Ox-A used at 0.3-1µM. Ox-A was first applied in control ACSF and then in BIC or TTX. *, p<0.05; **, p<0.01; ***, p<0.001; ns, not statistically significant, Bonferroni's *post-hoc* test. Black arrows: multiple treatments of the same neuron. Panel b, d and e: data are represented as means ± SEM, *n* refers to the number of recorded neurons and Source Data are provided as a Source Data file.

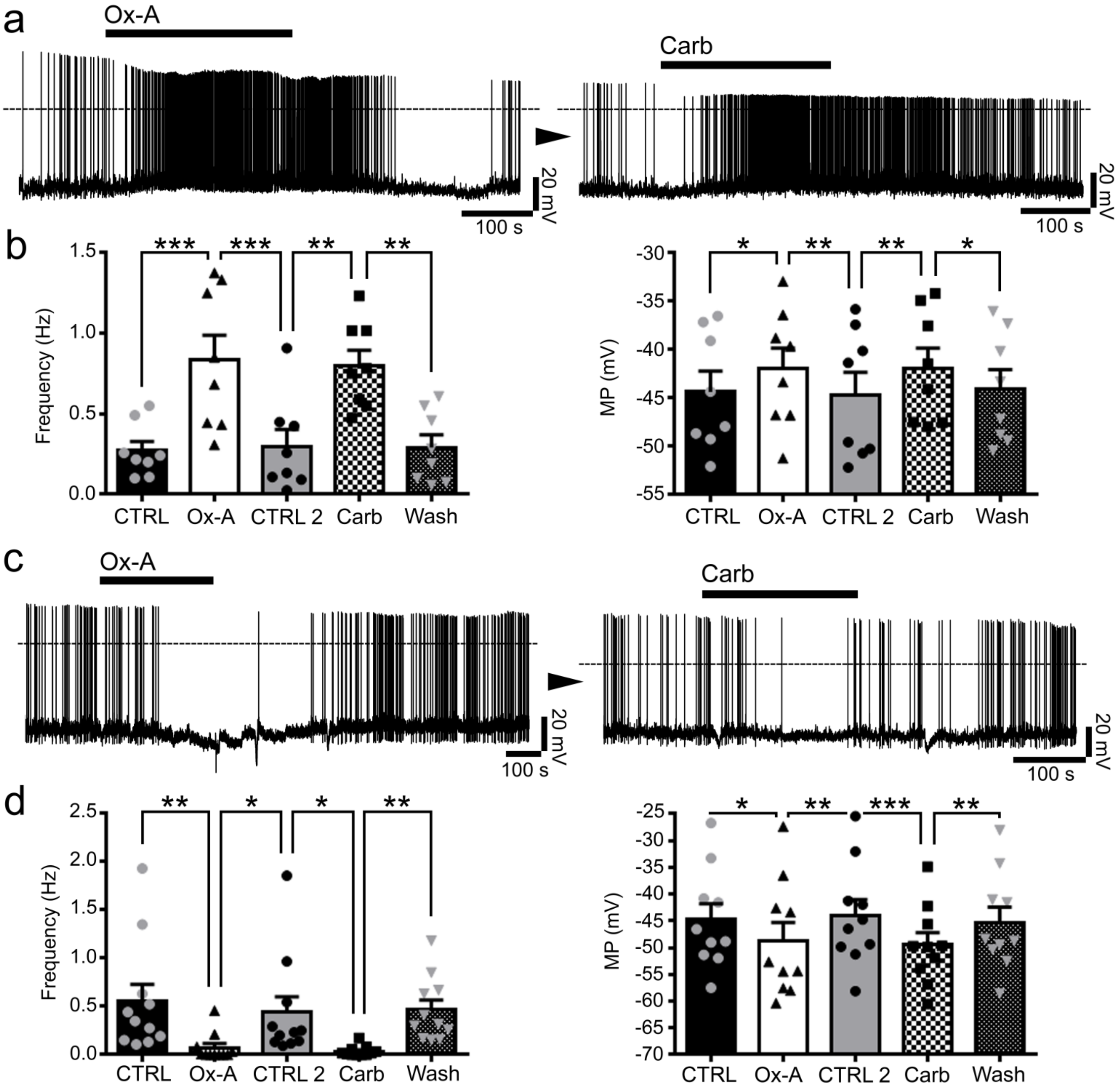

Suppl. Fig. 3. The VLPO neurons excited by orexin are also excited by carbachol and those inhibited, are also inhibited by carbachol. a-b) Ox-A (0.3-1μM) and carbachol (Carb; 50μM) excite 42% of VLPO neurons (*a*). Effects on the action potential frequency (*b left*; *n*=8; one-way ANOVA, $F_{(4, 35)}$=13.16; *p*<0.0001; CTRL vs Ox-A, *adj-p*=0.0003; Ox-A vs CTRL 2, *adj-p*=0.0006; CTRL 2 vs Carb, *adj-p*=0.0014; Carb vs Wash, *adj-p*=0.0011) and the membrane potential (*b right*; *n*=8; one-way ANOVA, $F_{(4, 35)}$=7.27;

p=0.0004; CTRL vs Ox-A, *adj-p*=0.0220; Ox-A vs CTRL 2, *adj-p*=0.0071; CTRL 2 vs Carb, *adj-p*=0.0051; Carb vs Wash, *adj-p*=0.0473). **c-d**) Ox-A and carbachol inhibit the remaining 58% of VLPO neurons (*c*). Effects on the action potential frequency (*d left*; n=11; one-way ANOVA, $F_{(4, 50)}$=8.13; p<0.0001; CTRL vs Ox-A, *adj-p*=0.0024; Ox-A vs CTRL 2, *adj-p*=0.0384; CTRL 2 vs Carb, *adj-p*=0.0165; Carb vs Wash, *adj-p*=0.0088) and the membrane potential (*d right*; n=10; one-way ANOVA, $F_{(4, 45)}$=9.63; p<0.0001; CTRL vs Ox-A, *adj-p*=0.0121; Ox-A vs CTRL 2, *adj-p*=0.0020; CTRL 2 vs Carb, *adj-p*=0.0002; Carb vs Wash, *adj-p*=0.0064). *, p<0.05, **, p<0.01, ***, p<0.001, Bonferroni's *post-hoc* test. Black arrows: multiple treatment of the same neuron. Panel b and d: data are represented as means ± SEM, n refers to the number of recorded neurons and Source Data are provided as a Source Data file.

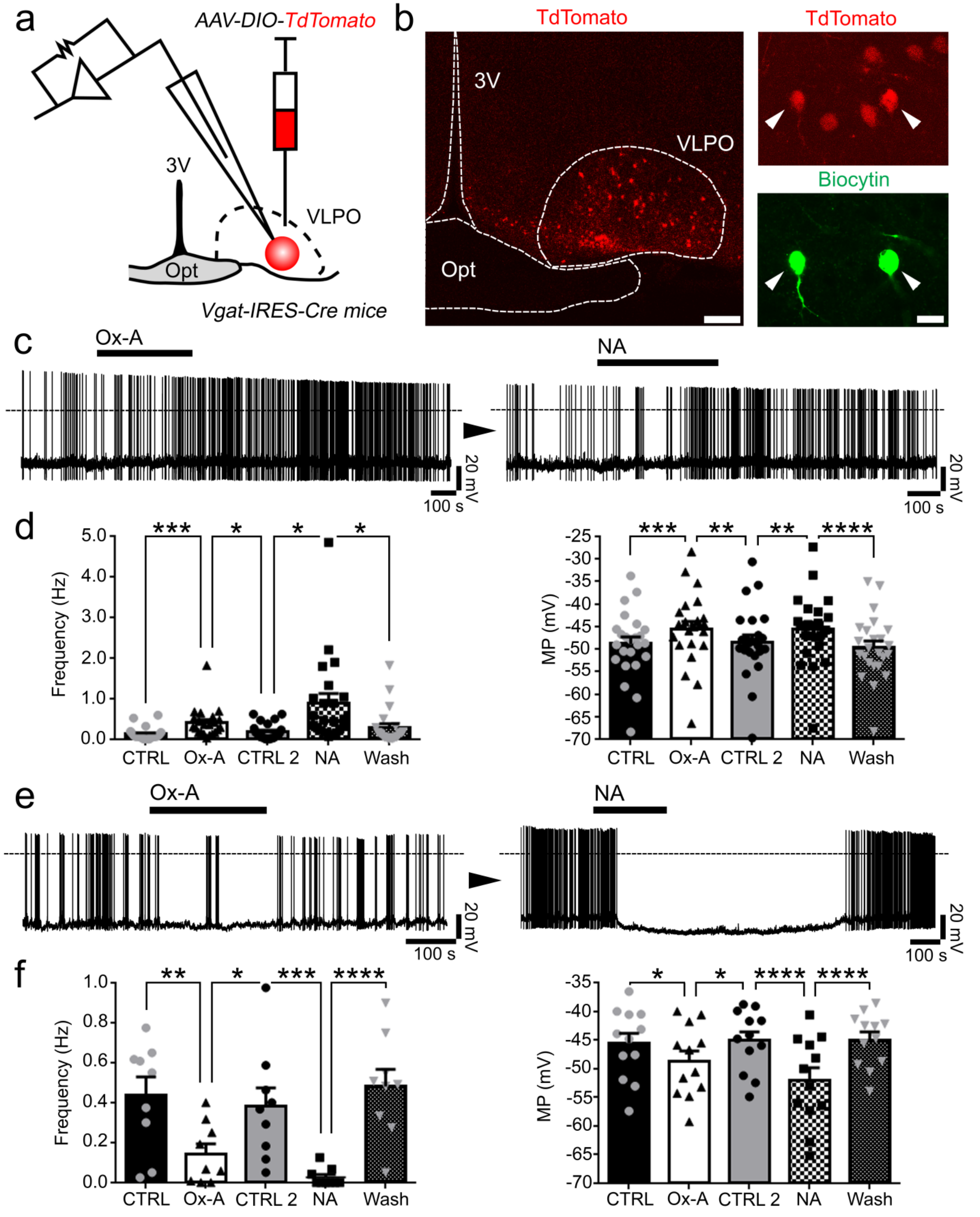

Suppl. Fig. 4. Dual response of orexin and noradrenalin in VLPO GABAergic neurons. a-b) We recorded from TdTomato labelled VLPO Vgat neurons in *Vgat-IRES-Cre* mice injected with *AAV-DIO-TdTomato* into the VLPO. Td-Tomato labeled neurons

at low magnification (*b left*; scale bar: 200µm) and at high magnification, showing two VLPO recorded neurons *post-hoc* labeled in green for biocytin (*b right*; scale bar: 20µm). **c-d**) VLPO Vgat neurons excited by Ox-A (1µM) are excited by NA (50µM) (*c*). Effects on action potential frequency (*d left; n*=22; one-way ANOVA, $F_{(4, 105)}$=10.90; *p*=0.0014; CTRL vs Ox-A, *adj-p*=0.0006; Ox-A vs CTRL 2, *adj-p*=0.0150; CTRL 2 vs NA, *adj-p*=0.0284; NA vs Wash *adj-p*=0.0119) and membrane potential (*d right*; *n*=24; one-way ANOVA, $F_{(4,115)}$=11.19, *p*<0.0001; CTRL vs Ox-A, *adj-p*=0.0008; Ox-A vs CTRL 2, *adj-p*=0.0048; CTRL 2 vs NA, *adj-p*=0.0084; NA vs Wash, *adj-p*<0.0001). **e-f**) VLPO Vgat neurons inhibited by Ox-A are inhibited by NA (*e*). Effects on action potential frequency (*f left; n*=9; one-way ANOVA, $F_{(4, 40)}$=14.03; *p*<0.0001; CTRL vs Ox-A, *adj-p*=0.0043; Ox-A vs CTRL 2, *adj-p*=0.0339; CTRL 2 vs NA, *adj-p*=0.0004; NA vs Wash, *adj-p*<0.0001) and membrane potential (*f right; n*=12; one-way ANOVA, $F_{(4, 55)}$=17.77; *p*<0.0001; CTRL vs Ox-A, *adj-p*=0.0429; Ox-A vs CTRL 2, *adj-p*=0.0117; CTRL 2 vs NA, *adj-p*<0.0001; NA vs Wash, *adj-p*<0.0001). *, *p*<0.05, **, *p*<0.01, ***, *p*<0.001, ****, *p*<0.0001, Bonferroni's *post-hoc* test. Black arrows: multiple treatments of the same neuron. 3V, third ventricle; Opt, optical chiasm. Panel d and f: data are represented as means ± SEM, *n* refers to the number of recorded neurons and Source Data are provided as a Source Data file.

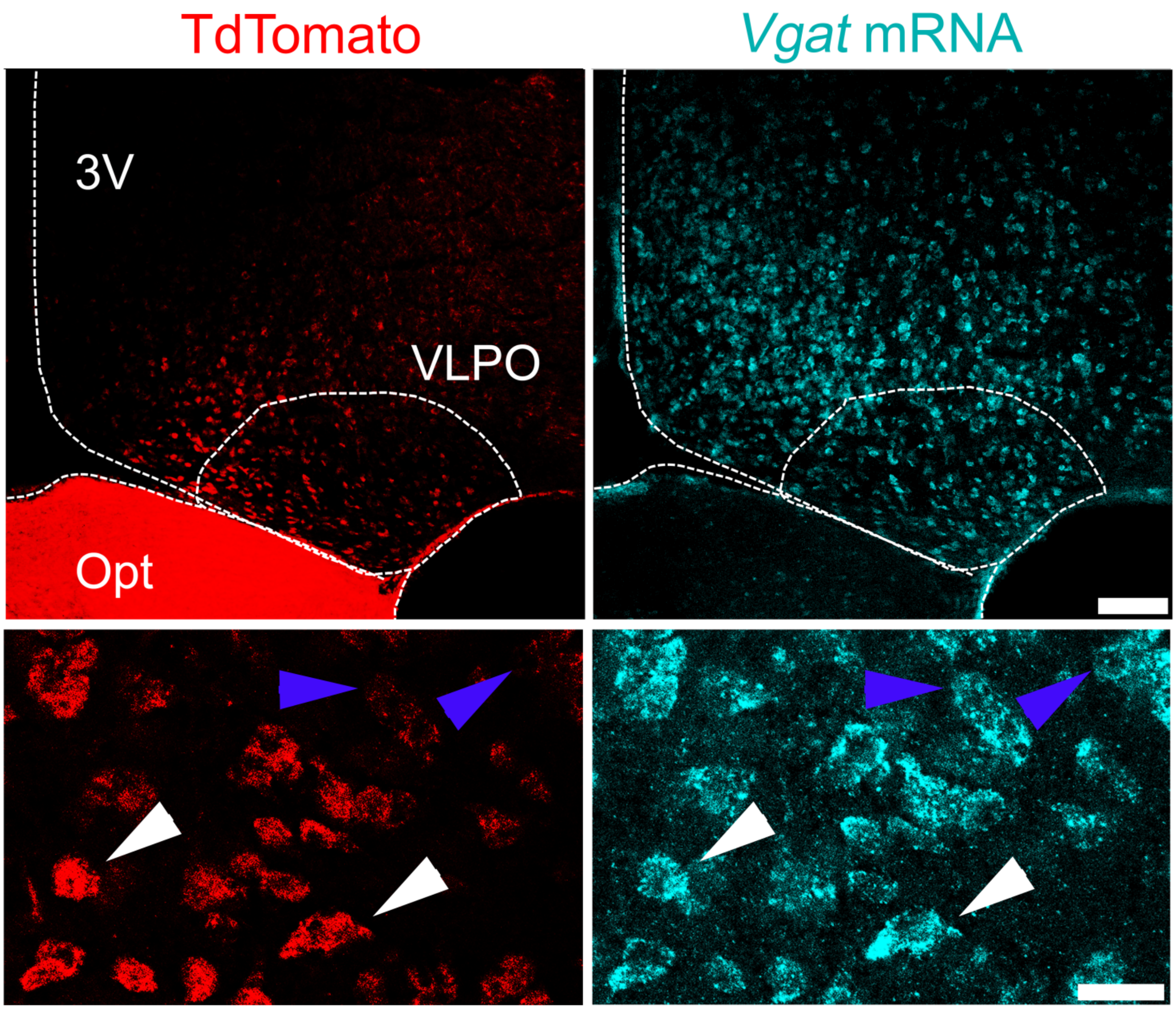

Suppl. Fig. 5. Histological assessment by *in situ hybridization* of *AAV-DIO-TdTomato* and *AAV-DIO-GFP* in the VLPO of *Vgat-IRES-Cre* mice. Both the AAV-DIO-TdTomato and the AAV-DIO-GFP selectively labeled Vgat neurons in the VLPO. Confocal images of TdTomato expressing neurons (immunolabeled in red) and DIG-labeled RNA probe *in situ hybridization* for *Vgat* mRNA (Cy5; far-red, pseudocolored in cyan) (*top*; scale bar: 100μm) and at higher magnification (*bottom*; white arrows: double labeled neurons; blue arrows: neurons expressing *Vgat* mRNA but not TdTomato; scale bar: 20μm). 3V, third ventricle; Opt, optical chiasm.

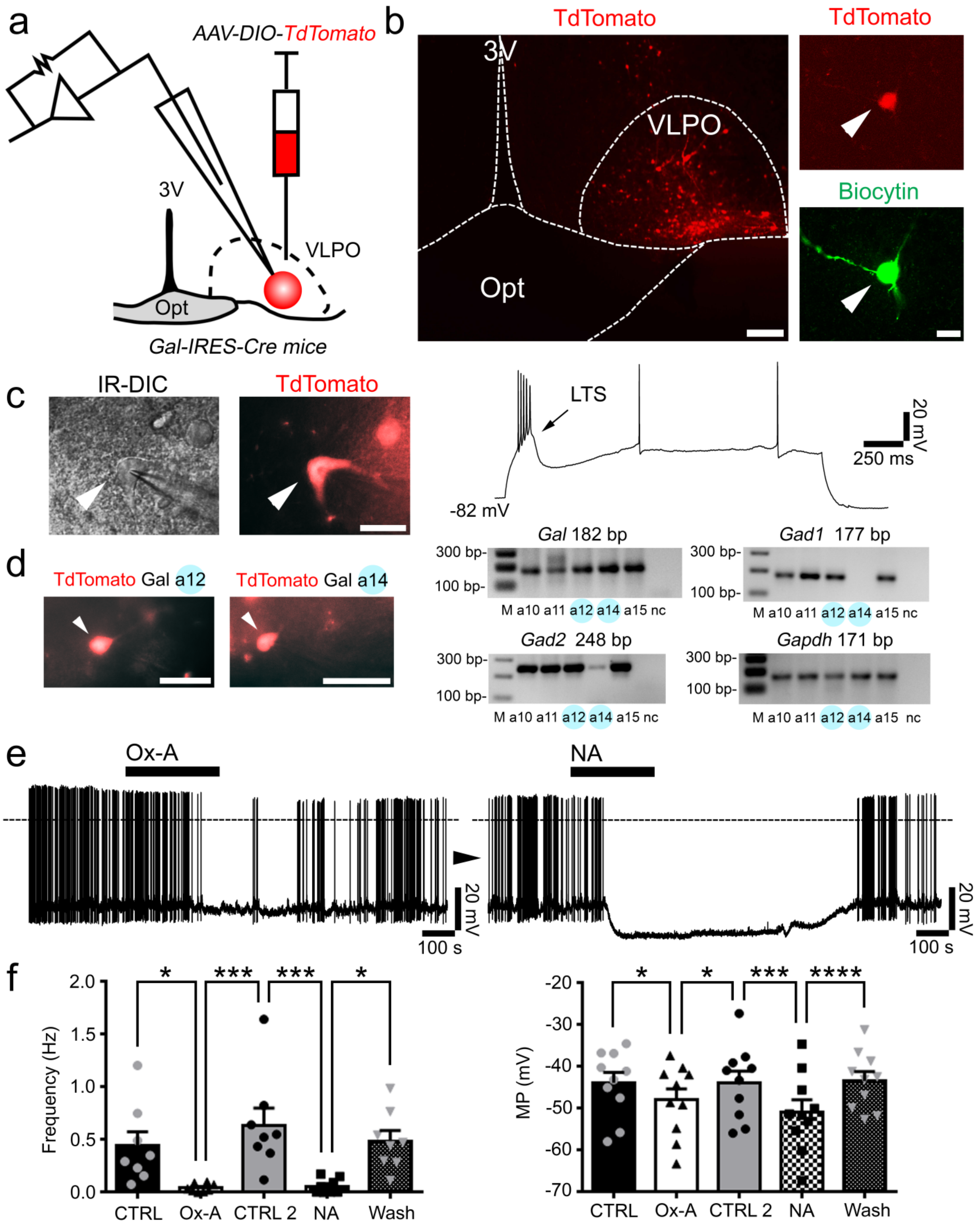

Suppl. Fig. 6. VLPO galanin neurons are inhibited by orexin and noradrenalin. a) We recorded from TdTomato labeled $VLPO^{GABA/Gal}$ neurons in *Gal-IRES-Cre* mice injected into the VLPO with *AAV-DIO-TdTomato.* **b**) Distribution of TdTomato

VLPO$^{GABA/Gal}$ neurons in a recording slice (*left*; scale bar: 200µm) and a recorded TdTomato VLPO$^{GABA/Gal}$ neuron after *post-hoc* staining for biocytin in green (*right*: scale bar: 20µm). **c-e**) TdTomato labeled VLPO$^{GABA/Gal}$ neurons have LTS (*C*), express galanin and GABAergic markers (*D*) and are inhibited by Ox-A (1µM; *E left*) and by NA (50µM; *E right*). Single cell RT-PCR for *galanin* (*Gal*; 182 bp), *Gad1* (177 bp), *Gad2* (248 bp) and *Gapdh* (171 bp; housekeeping gene) (*n*=5). Scale bars: 20µm in panel *C* and 50µm in panel *D*. **f**) Mean effects of Ox-A and NA on the action potential frequency (*F left*; one-way ANOVA, $F_{(4, 35)}$ = 9.34, *p*<0.0001; *n*=8; CTRL vs Ox-A, *adj-p*=0.0315; Ox-A vs CTRL 2, *adj-p*=0.0005; CTRL 2 vs NA, *adj-p*=0.0007; NA vs Wash, *adj-p*=0.0180) and the membrane potential (*F right*; one- way ANOVA, $F_{(4, 45)}$ = 11.51; *p*<0.0001; *n*=10; CTRL vs Ox-A, *adj-p*=0.0481; Ox-A vs CTRL 2, *adj-p*=0.0484; CTRL 2 vs NA, *adj-p*=0.0001; NA vs Wash, *adj-p*<0.0001) of VLPO$^{GABA/Gal}$ neurons. *, *p*<0.05, **, *p*<0.01, ***, *p*<0.001, ****, *p*<0.0001, Bonferroni's *post-hoc* test. 3V, third ventricle; Opt, optical chiasm. Panel f: data are represented as means ± SEM and *n* refers to the number of recorded neurons. Panel d and f: Source Data are provided as a Source Data file.

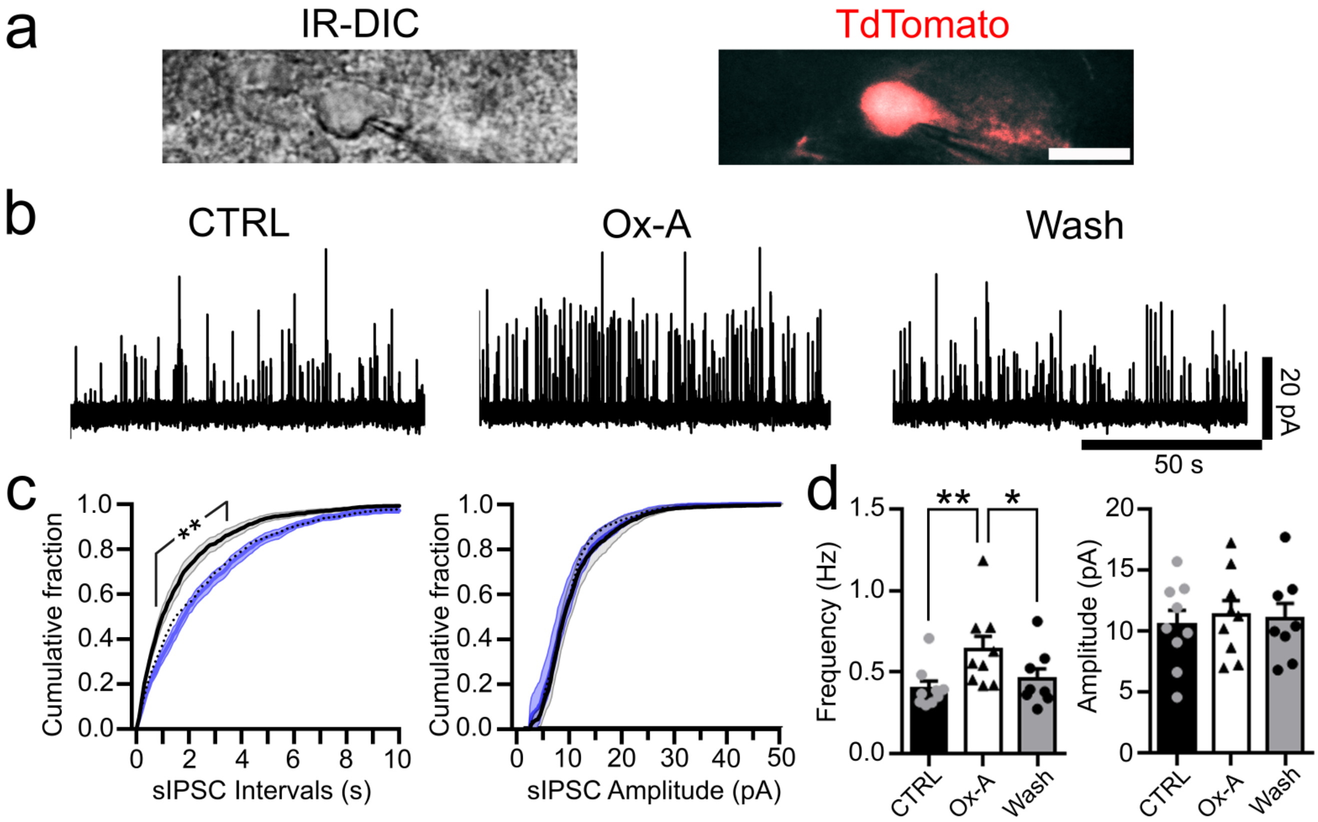

Suppl. Fig. 7. Orexin increases the GABAergic afferent input to VLPO$^{GABA/Gal}$ neurons. **a**) We recorded from VLPO$^{GABA/Gal}$ neurons labeled with TdTomato in *Gal-IRES-Cre* mice injected into the VLPO with *AAV-DIO-TdTomato* (scale bar: 20µm). **b**) Ox-A increases the sIPSC frequency in VLPO$^{GABA/Gal}$ neurons. **c**) Cumulative distribution plots of the sIPSC inter-event intervals (*left*; 100ms bins; two-way ANOVA, $F_{(2, 23)}$=5.934, *p*=0.0084; control vs Ox-A, *adj-p*=0.0012; Bonferroni's *post-hoc* test) and the sIPSC amplitudes (*right*; 0.25pA bins; two-way ANOVA, $F_{(1, 8)}$=1.46, *p*=0.2618; control vs Ox-A, *adj-p*>0.9999; Bonferroni's *post-hoc* test) compiled from 9 VLPO$^{GABA/Gal}$ neurons (blue: control; black: Ox-A; dotted: wash; light blue shaded area: control ± SEM and grey shaded area: Ox-A ± SEM). **d**) Mean sIPSC frequency (*left; n*=9, one-way ANOVA, $F_{(2, 23)}$=13.13, *p*=0.0016; CTRL vs Ox-A, *adj-p*=0.0067; Ox-A vs Wash, *adj-p*=0.0376) and sIPSC amplitude (*right; n*=9; one-way ANOVA, $F_{(2, 23)}$=0.80, *p*=0.4293) in control, Ox-A and washout. *, *p*<0.05 and **, *p*<0.01, Bonferroni's *post-hoc* test. sIPSC recorded at Vh=0mV, in kynurenic acid (1mM). Panel d: data are represented as means ± SEM and *n* refers to the number of recorded neurons. Panel c and d: Source Data are provided as a Source Data file.

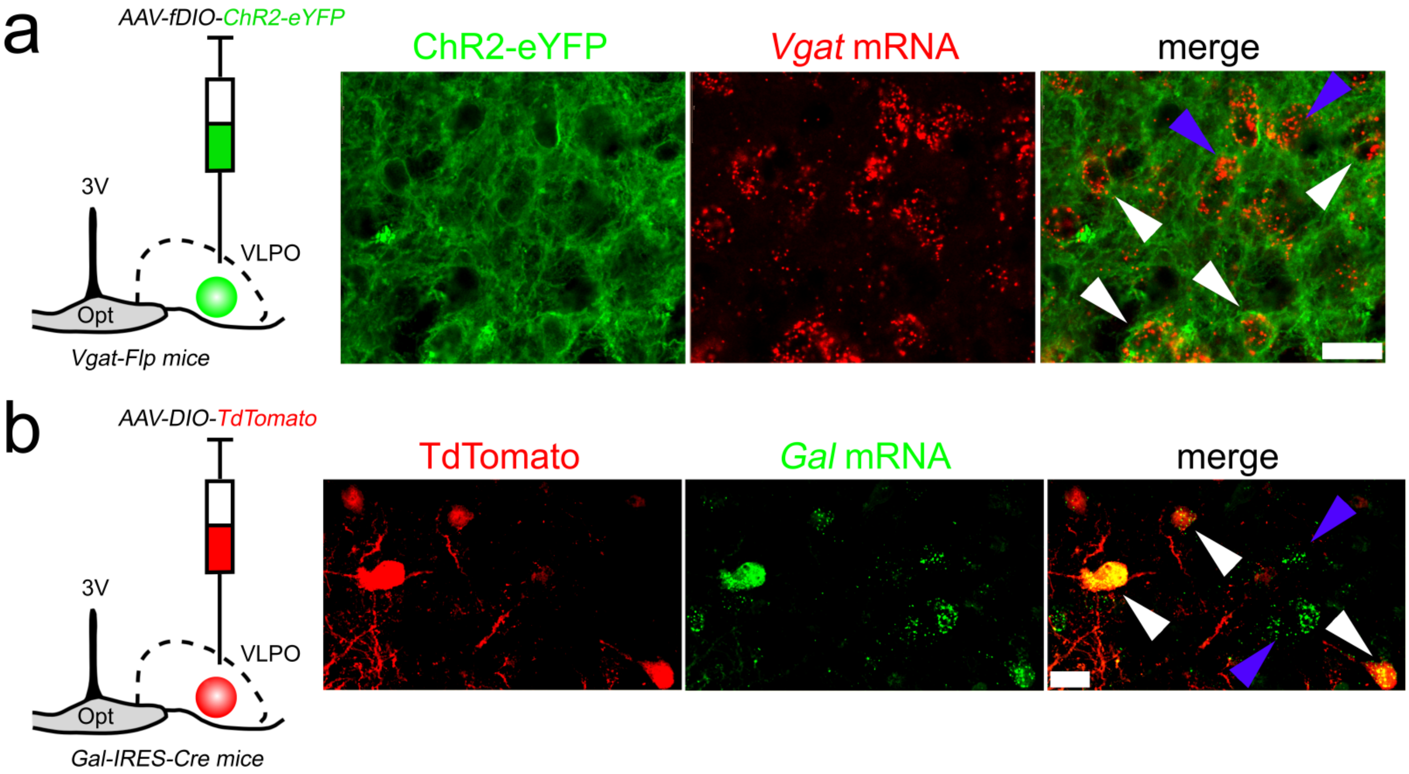

Suppl. Fig. 8. Histological assessment by RNA scope *in situ hybridization* of *AAV-fDIO-ChR2-eYFP* and *AAV- DIO-TdTomato* in VLPO of *Vgat-Flp* and *Gal-IRES-Cre* mice. a) The *AAV-fDIO-ChR2-eYFP* selectively labeled Vgatneurons in VLPO. Confocal images of ChR2-eYFP (green) and labeled RNA probes for *Vgat* (Cy3, red). **b**) The *AAV-DIO-TdTomato* selectively labeled galanin neurons in VLPO. Confocal images of TdTomato-expressing neurons and RNA probes for *galanin* (Fluorescein, green). White arrows: double labeled neurons; blue arrows: neurons expressing *Vgat* or *Gal* mRNAs but not YFP or TdTomato (scale bars: 20μm). 3V, third ventricle; Opt, optical chiasm.

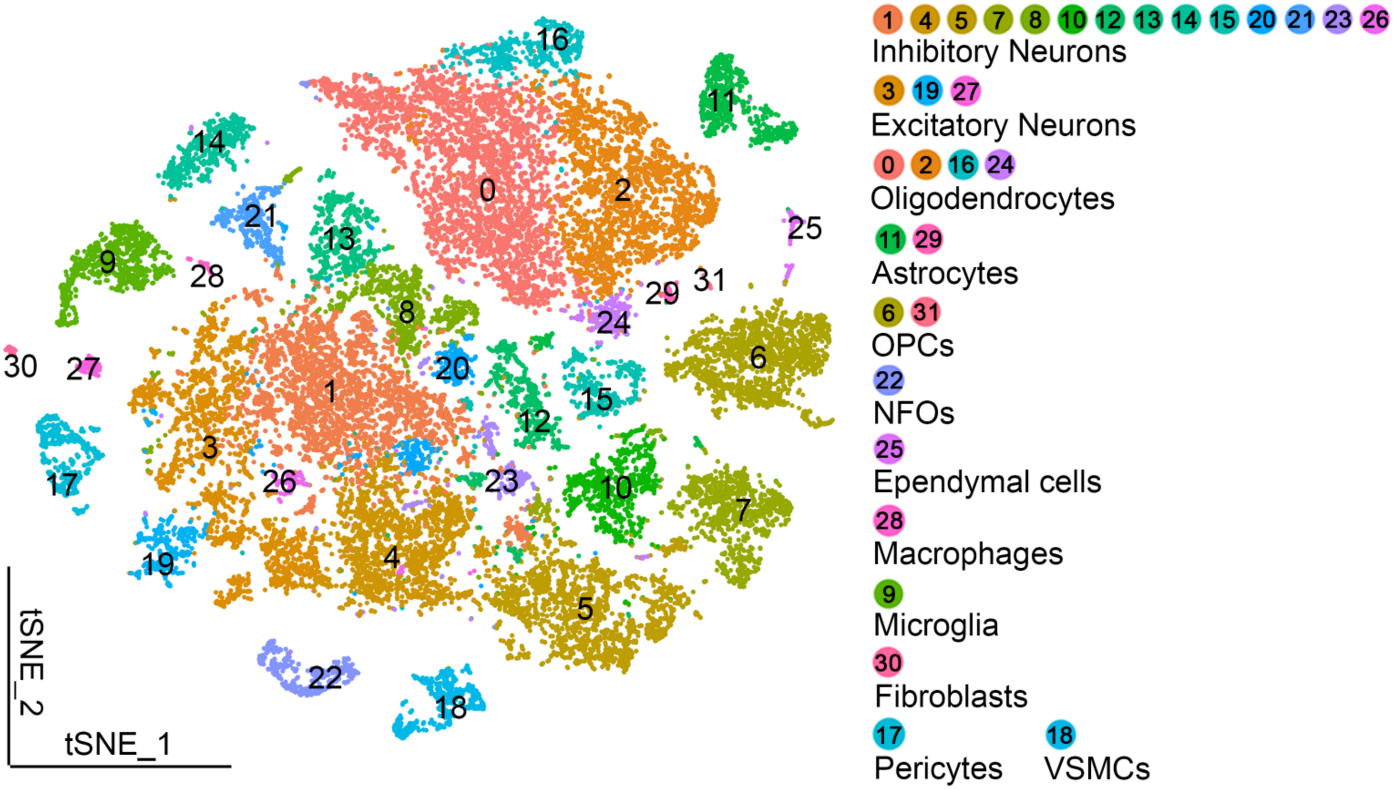

Suppl. Fig. 9. t-SNE plot of the POA. A two-dimensional t-SNE plot representing 28,041 cells profiled from the POA. Cells (dots) are color-coded by cluster membership (clusters 0-31) and labelled *post hoc* by cell types, marker analysis and anatomical distinctions (OPCs= oligodendrocyte precursor cells, NFOs= newly formed oligodendrocytes, VSMCs=Vascular smooth muscle cells). Source Data are provided as a Source Data file.

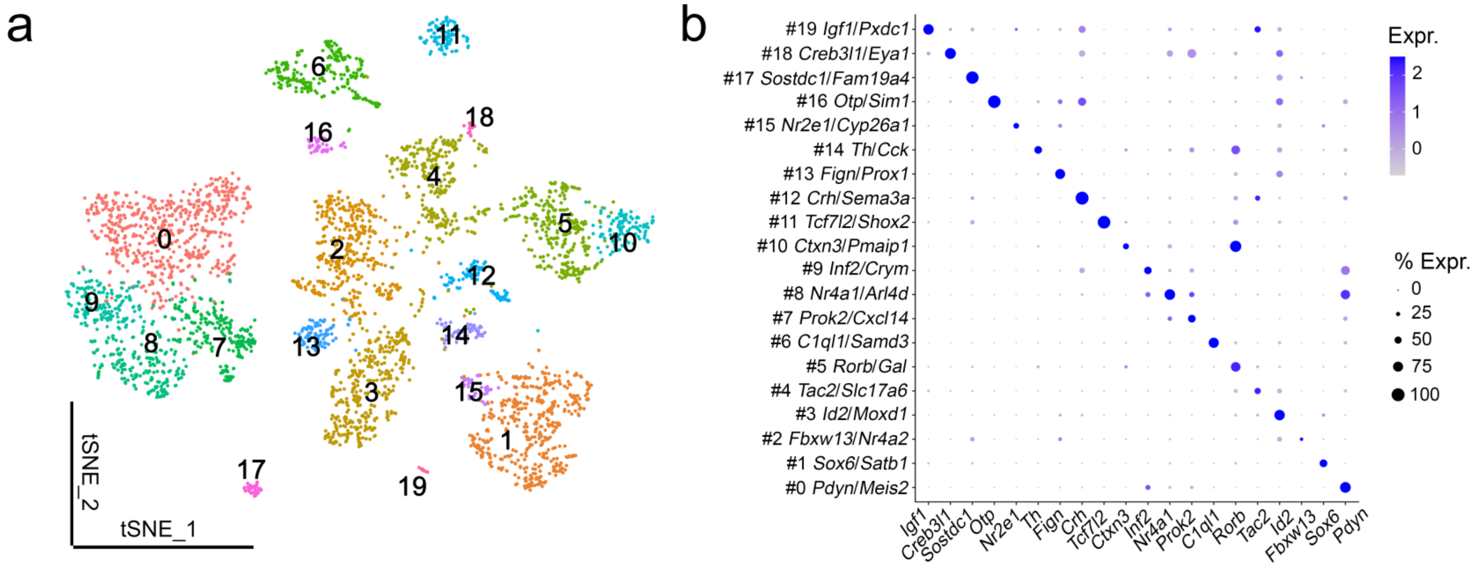

Suppl. Fig. 10. t-SNE and dotplot of VLPO neuronal clusters. a) A two-dimensional t-SNE plot representing 4,174 neurons profiled from the VLPO region. Neurons (dots) are color-coded by cluster membership (clusters 0-19) and clusters ID numbers are ordered based on the cluster size. **b**) Dot plot depicting the top marker gene (*x axis*) for each cluster (*y axis*). Dot size represents the percentage of neurons in a cluster that expresses a specific gene (*bottom right*). The color intensity indicates expression level (*top right*). We defined each cluster by the top 2 differentially expressed genes and plotted the most significant (*adj-p*<0.05). Test used: *Wilcoxon Rank Sum two-sided* Bonferroni-corrected Test. Source Data are provided as a Source Data file.

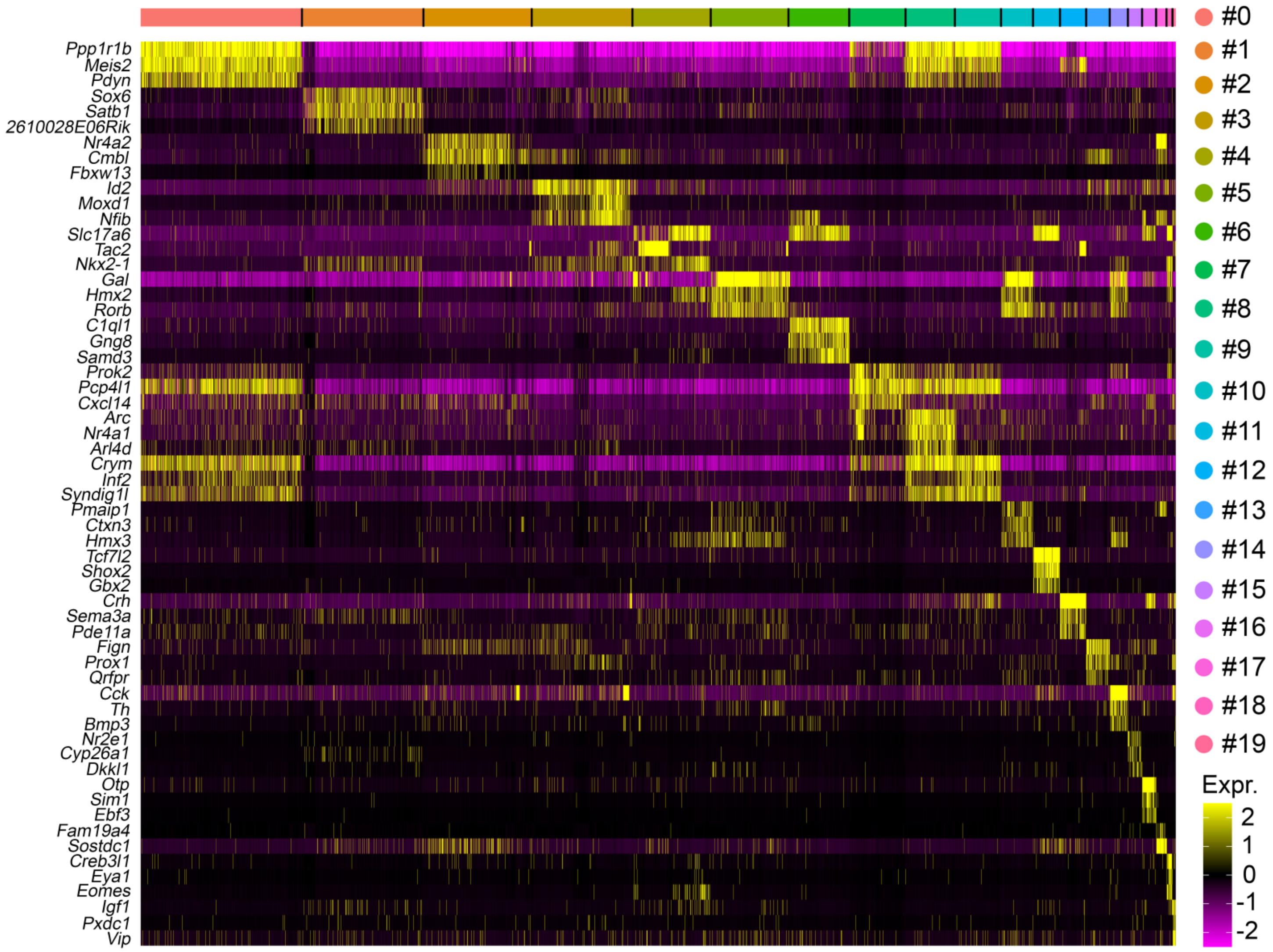

Suppl. Fig. 11. Heat map of the top 3 marker genes for each cluster of the VLPO region. Heat map showing the expression levels of the top 3 marker genes for each cluster found in the VLPO region. The clusters are represented by a color-coded bar on the *x axis* and genes are listed on the *y axis*. Expression levels are indicated by a scale bar (*bottom right*) and Clusters ID are listed in the legend as color dots (*top right*). Heat map expression values are represented as z-score. Source Data are provided as a Source Data file.

AAV	Inj. site	Mouse line	mice	% of transfected neurons that are Vgat (+), Gal (+) or Ox (+)	% of Vgat (+), Gal (+) or Ox (+) that are transfected
AAV-DIO-TdTomato	VLPO	*Vgat-IRES-Cre*	2	TdTomato (+) / Vgat mRNA ISH (+) 97.6% (*n* = 125)	Vgat mRNA ISH (+) / TdTomato (+) 38.2% (*n* = 319)
AAV-DIO-GFP	VLPO	*Vgat-IRES-Cre*	2	GFP (+) / Vgat mRNA ISH (+) 99.3% (*n* = 134)	Vgat mRNA ISH (+) / GFP (+) 27.6% (*n* = 482)
AAV-fDIO-ChR2-eYFP	VLPO	*Vgat-Flp*	2	ChR2-YFP (+) / Vgat mRNA ISH (+) 91.9 % (*n* = 234)	Vgat mRNA ISH (+) / ChR2-YFP (+) 32.4 % (*n* = 664)
AAV-DIO-TdTomato	VLPO	*Gal-IRES-Cre*	2	TdTomato (+) / Gal mRNA ISH (+) 92 % (*n* = 250)	Gal mRNA ISH (+) / TdTomato (+) 42.6 % (*n* = 540)
AAV-DIO-ChR2-eYFP	LH	*Ox-IRES-Cre*	2	ChR2-YFP (+) / Ox IHC (+) 96.0% (*n* = 223)	Ox IHC (+) / ChR2-YFP (+) 34.7% (*n* = 616)
AAV-DIO-ChR2-mCherry	LH	*Ox-IRES-Cre*	2	ChR2-mCherry (+) / Ox IHC (+) 94.6% (*n* = 168)	Ox IHC (+) / ChR2-mCherry (+) 36.4% (*n* = 437)

Suppl. Table 1. Histological assessment of the AAVs in *Cre* and *Flp* mouse lines. The specificity of the AAVs was quantified as the % of transduced neurons that expresses Vgat or Gal or Ox (% of neurons labeled by TdTomato, GFP, ChR2-YFP or ChR2-mCherry that expressed Vgat, Gal or Ox). The AAV transduction rate was quantified as the % of Vgat(+), Gal(+) or Ox(+) that are transduced (% of Vgat, Gal or Ox(+) neurons that were labeled by TdTomato, GFP, ChR2-YFP or ChR2-mCherry). ISH, *in situ* hybridization; IHC, immunohistochemistry; *n*, number of cells counted. Source Data are provided as Source Data file.

For Suppl. Tables 2-7 (please refer to the excel file)

Suppl. Table 2. Differential gene expression in the POA non-neuronal and neuronal clusters. Output from the *FindAllMarkers()* function built in Seurat. Differential gene expression (FoldChange) was performed for each gene between each cluster and all the other cells in the dataset (log FoldChange, *p* and *adj-p* and a pct.1 and pct.2). Log FoldChange represents the log-ratio of gene's expression values between two different conditions. With pct.1 as % of cells of a cluster expressing a specific gene and pct.2 as % of all the other cells in the dataset expressing that gene. *p* calculated using *Wilcoxon Rank Sum two-sided* Test. *Adj-p* is based on Bonferroni correction using all 3000 features (genes) in the dataset.

Suppl. Table 3. Differential gene expression in the VLPO neuronal clusters.

As in Suppl. Table 2. Test used: *Wilcoxon Rank Sum two-sided* Bonferroni-corrected Test.

Suppl. Table 4. Differential gene expression between VLPO^{GABA} (#0-3; 7-9; 12, 13, 15, 17, 19) and VLPO$^{GABA/Gal}$ clusters (#5, 10, 14).

As in Suppl. Table 2. Test used: *Wilcoxon Rank Sum two-sided* Bonferroni-corrected Test.

Suppl. Table 5. Differential gene expression between the VLPO^{GABA} cluster #1 and all combined VLPO$^{GABA/Gal}$ clusters (#5, 10, 14).

As in Suppl. Table 2. Test used: *Wilcoxon Rank Sum two-sided* Bonferroni-corrected Test.

Suppl. Table 6. Differential gene expression between the VLPO^{GABA} cluster #1 and all the other VLPO^{GABA} clusters (#0, 2, 3; 7-9; 12, 13, 15, 17, 19).

As in Suppl. Table 2. Test used: *Wilcoxon Rank Sum two-sided* Bonferroni-corrected Test.

Suppl. Table 7. Differential gene expression between the VLPO^{GABA} clusters (#0, 2, 3; 7-9; 12, 13, 15, 17, 19) and all combined VLPO$^{GABA/Gal}$ clusters (#5, 10, 14).

As in Suppl. Table 2. Test used: *Wilcoxon Rank Sum two-sided* Bonferroni-corrected Test.

mRNA probes	site	Mouse line	mice	% of Vgat(+) and Gal(-) that are Hcrtr2(+)	% of Vgat(+) and Gal(+) that are Hcrtr2(+)
Vgat, Gal, Hcrtr2	VLPO	WT	3	28.5% (*n* = 1717)	6.2% (*n* = 569)

Suppl. Table 8. *In situ* hybridization by RNA scope in VLPO. Percentage of the $VLPO^{GABA}$ neurons (Vgat(+) and Gal(-)) that expresses the Ox_2R (*Hcrtr2*) mRNA. Percentage of $VLPO^{GABA/Gal}$ neurons (Vgat(+) and Gal(+)) that expresses *Hcrtr2* mRNA. Source Data are provided as Source Data file.
