# Supplementary material for: Orexin neurons inhibit sleep to promote arousal
Source: Nat Commun. 2022 Jul 18;13:4163. doi: 10.1038/s41467-022-31591-y (PMC9293990; doi:10.1038/s41467-022-31591-y)
Supplement: Supplementary file 4 — Reporting Summary [file 41467_2022_31591_MOESM4_ESM.pdf]

nature portfolio

Corresponding author(s): Elda Arrigoni

Last updated by author(s): May 26, 2022

Reporting Summary

Nature Portfolio wishes to improve the reproducibility of the work that we publish. This form provides structure for consistency and transparency in reporting. For further information on Nature Portfolio policies, see our Editorial Policies and the Editorial Policy Checklist.

Statistics

For all statistical analyses, confirm that the following items are present in the figure legend, table legend, main text, or Methods section.

n/a	Confirmed	
☐	☒	The exact sample size (*n*) for each experimental group/condition, given as a discrete number and unit of measurement
☐	☒	A statement on whether measurements were taken from distinct samples or whether the same sample was measured repeatedly
☐	☒	The statistical test(s) used AND whether they are one- or two-sided<br>*Only common tests should be described solely by name; describe more complex techniques in the Methods section.*
☐	☒	A description of all covariates tested
☐	☒	A description of any assumptions or corrections, such as tests of normality and adjustment for multiple comparisons
☐	☒	A full description of the statistical parameters including central tendency (e.g. means) or other basic estimates (e.g. regression coefficient) AND variation (e.g. standard deviation) or associated estimates of uncertainty (e.g. confidence intervals)
☐	☒	For null hypothesis testing, the test statistic (e.g. *F*, *t*, *r*) with confidence intervals, effect sizes, degrees of freedom and *P* value noted<br>*Give P values as exact values whenever suitable.*
☒	☐	For Bayesian analysis, information on the choice of priors and Markov chain Monte Carlo settings
☒	☐	For hierarchical and complex designs, identification of the appropriate level for tests and full reporting of outcomes
☒	☐	Estimates of effect sizes (e.g. Cohen's *d*, Pearson's *r*), indicating how they were calculated

Our web collection on statistics for biologists contains articles on many of the points above.

Software and code

Policy information about availability of computer code

Data collection	For sleep/wake data collection: MATLAB (version R2020B; Mathworks) and Spike 2 (Cambridge Electronic Design) software;<br>For in vitro electrophysiology data collection: Clampex 9.0 (Molecular Devices) software;<br>For image (histology) data collection: OlyVIA (Olympus) and Zen 2009 (Zeiss) software;<br>For scRNA-seq data collection: no software was used.
Data analysis	For sleep/wake data analysis: Sigmastat (version 4.0, Systat Software Inc. San Jose, CA) and MATLAB (version R2020B; Mathworks);<br>For in vitro electrophysiology data analysis: Clampfit 10 (Molecular Devices), MiniAnalysis 6 (Synaptosoft, Leonia, NJ), Python 3 (www.python.org) and MATLAB (version R2020B; MathWorks) software;<br>For image (histology) data analysis: OlyVIA (Olympus), Zen 2009 (Zeiss) and Image J software;<br>For scRT-PCR data analysis: Primer 3web 4.1.0 (http://primer3.wi.mit.edu/) and Genetyx software;<br>For scRNA-seq data analysis: Seurat (https://satijalab.org/seurat/; version 3.1.5) and all its dependencies as listed in the README file. The R version used was 3.6.3.<br>For figure preparation: Igor Pro 6 (WaveMetrics), Prism 7 (GraphPad, La Jolla, CA), Inkscape (GitLab) and Photoshop (Adobe) and BioRender software.

For manuscripts utilizing custom algorithms or software that are central to the research but not yet described in published literature, software must be made available to editors and reviewers. We strongly encourage code deposition in a community repository (e.g. GitHub). See the Nature Portfolio guidelines for submitting code & software for further information.

Data

Policy information about availability of data

All manuscripts must include a data availability statement. This statement should provide the following information, where applicable:

- Accession codes, unique identifiers, or web links for publicly available datasets
- A description of any restrictions on data availability
- For clinical datasets or third party data, please ensure that the statement adheres to our policy

Dataset from Moffit et. al. 2018 useful to reproduce all POA cell clustering is public. For scRNA-seq raw data, the accession code on the GEO repository is GSE113576 (Moffit et. al. 2018). For scRNA-seq analysis described in this study we provided expression count matrix, barcodes and gene IDs for POA and VLPO at the following link: https://doi.org/10.5281/zenodo.6570978. There is no restriction to scRNA-seq data availability.

Field-specific reporting

Please select the one below that is the best fit for your research. If you are not sure, read the appropriate sections before making your selection.

☒ Life sciences ☐ Behavioural & social sciences ☐ Ecological, evolutionary & environmental sciences

For a reference copy of the document with all sections, see nature.com/documents/nr-reporting-summary-flat.pdf

Life sciences study design

All studies must disclose on these points even when the disclosure is negative.

Sample size	Sample size is reported in the result section and in the figure legends. No method was used to estimate the sample size. Sample size was determined considering the biological variability and the distribution of the data obtained and analyzed for each experimental session.
Data exclusions	1) Behavioral recordings (sleep/wake analysis): data were excluded if the conditions of the histological validation were not met (i.e., cases in which there was not adequate bilateral transduction of the viral vector or optical fibers were not correctly positioned).<br>2) Electrophysiological recordings: neurons showing changes in input resistance of more than 10% over time, were excluded from the analysis.<br>3) Immunohistochemistry, in situ hybridization and RNA scope in situ hybridization: sections were excluded on the basis of tissue quality and non specific background labeling.<br>4) Single cell RT-PCR and RT-sqPCR: we considered scRT-PCR- and scRT sqPCR-negative and so to be excluded, samples in which Gapdh expression was undetectable.<br>5) Single cell RNA sequencing data analysis. Cells were excluded if the following criteria were not met: i) cells expressing >200 and <6000 genes and with a mitochondrial gene expression rate <10%. Genes were excluded if they were not detected in >2 cells.
Replication	Data were replicated in independent neurons or mice as indicated in the manuscript (see Figure Legends). Replications were all successful.
Randomization	1) Behavioral recordings (sleep/wake analysis): animals were randomly assigned to the behavioral groups during the experiment in which the opto-stimulation consisted of light pulses (5ms) delivered at frequencies 1, 5, 10, 20 Hz over a 10 s period, delivered in a random order throughout the recording session.
Blinding	1) Behavioral recordings (sleep/wake analysis): all behavioral recordings were scored by an investigator that was blinded to the recording conditions. 2) In vitro electrophysiological recordings: all data were analyzed either by automated software or by multiple investigators who were blind to the experimental conditions and to group allocation during data collection.

Reporting for specific materials, systems and methods

We require information from authors about some types of materials, experimental systems and methods used in many studies. Here, indicate whether each material, system or method listed is relevant to your study. If you are not sure if a list item applies to your research, read the appropriate section before selecting a response.

Materials & experimental systems

n/a	Involved in the study	
☐	☒	Antibodies
☒	☐	Eukaryotic cell lines
☒	☐	Palaeontology and archaeology
☐	☒	Animals and other organisms
☒	☐	Human research participants
☒	☐	Clinical data
☒	☐	Dual use research of concern

Methods

n/a	Involved in the study	
☒	☐	ChIP-seq
☒	☐	Flow cytometry
☒	☐	MRI-based neuroimaging

Antibodies

Antibodies used	rabbit anti-ds Red primary antibodies (1:3000: Catalog #: 632496; Lot #: 1904182; Takara Bio USA, Mountain View, CA);<br>chicken anti-GFP primary antibodies (1:2000; Catalog #: A10262; Lot #: 2156242; Invitrogen);<br>goat anti-Orexin-A primary antibodies (1:500; Catalog #: sc-8070; Lot #: A2915; Santa Cruz Biotechnology, Dallas, TX);<br>peroxidase-conjugated DIG primary antibodies (1:500, Catalog #: 11207733910; Lot #: 13296300; Roche Applied Science, now Millipore sigma);<br><br>streptavidin-conjugated Alexa Fluor-488 (1:500; Catalog #: S32354 and Lot#: 1719656);<br>streptavidin-conjugated Alexa Fluor-555 (1:500; Catalog #: S21381 and Lot#: 1010095);<br><br>Alexa Fluor-488-conjugated donkey anti-goat (1:500, Catalog #: A11055; Lot #: 1942238; Invitrogen) secondary antibodies;<br>Alexa Fluor-555-conjugated donkey anti-rabbit (1:500, Catalog #: A31572; Lot #: 2286312; Invitrogen) secondary antibodies;<br>Alexa Fluor-488-conjugated goat anti-chicken (1:500; Catalog #: A11039; Lot #: 2304258; Invitrogen) secondary antibodies;<br>Alexa Fluor-555-conjugated donkey anti-goat (1:500, Catalog #: A21432; Lot #: 1697092; Invitrogen) secondary antibodies;<br>Alexa Fluor-488-conjugated rabbit anti-chicken (1:500; Catalog #: NB710-94968; Lot #: 143-090; Novus Biologicals, Littleton, CO) secondary antibodies.
Validation	For validation see:<br>Takara Bio (rabbit anti-ds Red primary antibodies) https://www.takarabio.com/products/antibodies-and-elisa/fluorescent-protein-antibodies/red-fluorescent-protein-antibodies<br>Invitrogen (chicken anti-GFP) https://www.fishersci.com/shop/products/anti-green-fluorescent-protein/a10262<br>Santa Cruz Biotechnology (goat anti-Orexin-A) https://www.citeab.com/antibodies/820733-sc-8070-orexin-a-antibody-c-19<br>Roche (peroxidase-conjugated DIG) https://www.bioz.com/result/anti%20digoxigenin%20hrp%20conjugate/product/Roche<br>For secondary antibody Alexa Fluor-conjugated see Invitrogen and Novus Biologicals

Animals and other organisms

Policy information about studies involving animals; ARRIVE guidelines recommended for reporting animal research

Laboratory animals	Mice were treated in accordance with guidelines from the National Institute of Health Guide for the Care and Use of Laboratory Animals. All protocols were approved by Beth Israel Deaconess Medical Center Institutional Animal Care and Use Committee and all efforts were directed to minimize the number of animals and their suffering.<br>Adult mice, 6-12 weeks of age and an equal proportion of male and female mice, were used.<br>We used: 55 C57BL/6J (WT); 33 Vgat-IRES-Cre; 25 Galanin-IRES-Cre (Gal-IRES Cre); 11 Orexin-IRES-Cre (Ox-IRES-Cre) and 10 Vgat-Flp::Gal-IRES-Cre mice.<br>- WT mice were purchased from The Jackson Laboratory (Jax, Bar Harbor, ME);<br>- Vgat-IRES-Cre mice (Jax Slc32a1tm2(cre)Lowl/J, Cat. #016962);<br>- Gal-IRES-Cre mice were given to us by Dr. Ramalingam Vetrivelan (Neurology Department, BIDMC, Boston, MA);<br>- Vgat-Flp::Gal-IRES-Cre mice were obtained by crossing the Slc32a1 tm1.1(flpo)Hze mice (Vgat-Flp; Jax B6.Cg-Slc32a1tm1.1(flpo)Hze/J, Cat. #029591) with the Gal-IRES-Cre mice;<br>- Ox-IRES-Cre mice were generated, validated, and kindly provided by Drs. D.K., T.M., T.E.S. and B.B.L.;<br>-Ai14 cre reporter mice (Jax B6;129S6-Gt(ROSA)26Sortm14(CAG-tdTomato)Hze/J, Cat. #007908). These mice were used to generate the Ox-IRES-Cre mice. Detailed description and characterization of this mouse line will be reported separately (Howard et al., unpublished). Please contact D.K. and B.B.L. for info regarding number, sex and age. These mice were not directly used in this current study.
Wild animals	No wild animals were caught, used or obtained
Field-collected samples	No field-collected samples were caught, used or obtained
Ethics oversight	Mice were treated in accordance with guidelines from the National Institute of Health Guide for the Care and Use of Laboratory Animals. All protocols were approved by Beth Israel Deaconess Medical Center Institutional Animal Care and Use Committee and all efforts were directed to minimize the number of animals and their suffering.

Note that full information on the approval of the study protocol must also be provided in the manuscript.
